# Prospects and Limitations of Bioprinting in Studying Human Cells’ Responses to Extreme Environments

**DOI:** 10.3390/bioengineering13040458

**Published:** 2026-04-14

**Authors:** Taieba Tuba Rahman, Zhijian Pei, Hongmin Qin, Hamid R. Parsaei

**Affiliations:** 1 Department of Industrial & Systems Engineering, Texas A&M University, College Station, TX 77843, USA; zjpei@tamu.edu (Z.P.); hamid.parsaei@tamu.edu (H.R.P.); 2 Department of Biology, Texas A&M University, College Station, TX 77843, USA; hqin@tamu.edu

**Keywords:** bioprinting, extreme environment, human cells’ response

## Abstract

Understanding human’s responses to extreme environments holds significant importance for space exploration, deep-sea research, and environmental adaptation. Traditionally, human subjects were used to study humans’ responses to extreme environments. The main limitations of this approach include the inability to independently investigate specific cellular mechanisms, ethical and safety constraints, limited experimental controllability, and inter-individual variability that complicates mechanistic interpretation. Another approach is to study humans’ responses at the cellular level using 2D culture. This approach often exhibits limited reproducibility due to its inability to recapitulate physiologically relevant microenvironments. Bioprinting can enable studies on human’s responses at the cellular level and within 3D environments. One way is to study human cells’ responses to localized and transient extreme environments created during printing. Another way is to expose 3D printed samples (embedded with human cells) to extreme environments. However, the literature does not contain comprehensive review papers to discuss the prospects and limitations of bioprinting for investigating human cells’ responses to extreme environments. This review paper aims to fill this gap in the literature. It begins with a brief description of the effects of extreme environments on human health and summarizes reported studies on cells’ responses to extreme environments. Afterward, it discusses the prospects and limitations of the two ways of using bioprinting to investigate cells’ responses to extreme environments. Finally, it concludes with identifying knowledge gaps and proposing research directions in the application of bioprinting to study human cells’ responses to extreme environments.

## 1. Introduction

Exposure to extreme environments (such as high or low temperature, high pressure, and microgravity) can lead to a variety of adverse effects on human health depending on the nature and exposure duration of the extreme environment. Extreme heat exposure may result in dehydration, heat exhaustion, and potentially life-threatening heat stroke [1,2,3], whereas prolonged cold exposure can impair neurological function, reduce metabolic activity, and progress to hypothermia as thermoregulatory mechanisms fail [4]. Hypobaric environments occurring at high altitude reduce oxygen availability and may cause hypoxia, acute mountain sickness, and severe complications such as high-altitude pulmonary edema and cerebral edema [5,6,7,8,9,10,11,12]. Hyperbaric exposure during underwater or pressurized operations increases the risk of barotrauma, nitrogen narcosis, and decompression injury associated with rapid pressure changes [13,14,15,16,17]. Additionally, exposure to microgravity during space missions contributes to musculoskeletal deconditioning, bone density loss, and cardiovascular adaptation [18]. Table 1 summarizes several reported studies on human body’s responses to extreme environments. In these reported studies, human subjects were used to provide critical insight into humans’ responses at the organ or whole-body levels. The main limitation of this approach is the inability to independently investigate specific cellular mechanisms.

Since humans’ responses to extreme environments ultimately originate from the cellular level, it is important to understand how human cells respond to extreme environments. Another approach is to study humans’ responses at the cellular level using 2D culture. Table 2 summarizes several reported studies on cells’ responses to extreme environments using 2D cell culture [21,22,23]. Conventional 2D cell culture systems lack native tissue architecture, extracellular matrix interactions, and three-dimensional mechanical constraints that critically influence cellular behavior under stress [24]. As a result, cells’ responses observed in 2D cultures may not accurately reflect those occurring within tissues that preserve native architecture.

Several review papers have summarized the effects of individual extreme environments (such as heat stress [26,27,28], hypoxia [29], high-pressure environment [30], and microgravity [30,31,32]) on cellular behavior and human health. However, the existing review papers primarily cover reported studies based on conventional cell culture and animal models. None of them was focused on the prospects and limitations of using 3D printing to study human cells’ responses to extreme environments. This paper is intended to fill this gap in the literature.

In this review paper, Section 2 discusses the prospects and limitations of using 3D printing to investigate human cells’ responses to localized and transient extreme environments created during printing. Section 3 discusses the prospects and limitations of using 3D printing to investigate human cells’ responses to extreme environments with 3D printed samples containing human cells. Finally, Section 4 provides concluding remarks.

## 2. Investigation of Cells’ Responses to Localized and Transient Extreme Environments Created During Printing

### 2.1. Creation of Localized and Transient Extreme Environments During Printing

Bioprinting techniques can create localized and transient extreme environments during the printing process. In these localized and transient extreme environments, cells embedded in the bioink (mostly hydrogel) experience localized and transient mechanical, thermal, and radiative stresses. The type and magnitude of stresses experienced by cells depend on the printing technique and can be controlled through changes in printing parameters. Table 3 shows several types of localized and transient environmental stress created during printing.

In extrusion-based 3D printing, cells embedded in bioinks primarily experience shear stress during material extrusion. In this technique, cell-laden bioink is continuously extruded through a nozzle, and cells experience localized mechanical stresses, mainly shear stress [33]. Beyond mechanical stress, extrusion-based 3D printing can also introduce thermal and radiative stresses depending on the bioink type and crosslinking approach. Extrusion bioprinting commonly relies on hydrogel-based bioinks to maintain structural stability, which requires a crosslinking mechanism. Depending on the bioink type, crosslinking may occur through photocrosslinking, using ultraviolet or visible light, or through thermal crosslinking, involving temperature-induced gelation [34]. When temperature-sensitive bioinks such as collagen are used, thermal stress arises from temperature changes required to control their phase behavior during printing and gelation. Collagen-based bioinks are typically maintained at low temperatures (≈4–10 °C) to remain in a liquid state prior to printing, and after printing, are exposed to physiological temperatures (≈37 °C) to induce gelation [35]. This temperature transition creates a transient thermal gradient around the encapsulated cells, which can induce thermal stress [36]. In addition, when photocrosslinkable bioinks are employed, UV exposure, used for crosslinking, may generate radiative stress on cells.

In inkjet-based 3D printing, cells experience mechanical stress during droplet formation and ejection, with thermal inkjet systems potentially causing brief temperature fluctuations [37].

In stereolithography-based 3D printing, cells are exposed to ultraviolet or visible light during layer-by-layer photopolymerization, which primarily induces radiative stress [38].

In laser-assisted bioprinting, bioink droplets are transferred onto a substrate using focused laser pulses without nozzle contact. In this technique, cells are exposed to localized energy input during the printing process, which can introduce radiative and thermal stresses [39].

**Table 3:** Types of localized and transient environmental stress created during printing.

Bioprinting Technique	Type of Stress	Controlling Printing Parameter	Reference
Extrusion-based	Shear stress	Nozzle shape	[40,41]
		Nozzle diameter	[41,42]
		Nozzle length	
		Extrusion pressure	[41,42,43,44]
	Thermal stress	Printing temperature	[41]
	Thermal and radiative stress	UV exposure duration, UV intensity	[41]
Inkjet-based	Shear stress	Applied voltage, frequency, pulse duration	[45]
	Thermal stress	Nozzle temperature	[37]
Laser-assisted	Shear stress	Jetting speed due to bubble expansion	[46]
	Thermal and radiative stress	Laser exposure, laser intensity	[39,47]
Stereolithography	Radiative stress	UV exposure duration, UV intensity,	[38]
		Photoinitiator	

### 2.2. Variaion in the Intensity of Localized and Transient Extreme Emvironments by Changing Process Parameters

By changing printing process parameters, the intensity of the microenvironmental stress experienced by cells can be varied, which enables the systematic investigation of cells’ stress responses. For example, Rahman et al. investigated the effects of extrusion pressure on human bronchial epithelial (HBE) cells encapsulated in 3D printed samples [48]. Extrusion pressures up to 100 kPa were applied to evaluate how acute mechanical loading during printing influenced cell viability. These pressure levels exceed those typically experienced during normal breathing (≤2.9 kPa) [49,50] or coughing (~19.6 kPa) [51], and therefore serve as controlled mechanical challenges rather than representations of sustained environmental pressure exposure. Representative studies on cells’ responses to localized and transient extreme environments created during 3D printing are summarized in Table 4.

Reported studies have shown that in extrusion-based 3D printing, reducing nozzle diameter below 400 µm increases shear stress during extrusion, which can lead to reduced cell viability [41,42]. Increasing nozzle length above 8.9 mm increases the duration of shear exposure, causing cells to experience shear stress for a longer period during extrusion, which also contributes to reduced cell viability [44]. Besides nozzle geometry, increasing extrusion pressure above 0.5 bar intensifies shear-induced cellular stress, which causes membrane damage and reduced cell viability [42,43]. Shear stress can modulate signaling pathways associated with proliferation, migration, and the metabolism of cells, highlighting its broader impact on cellular function [54]. In addition, shear stress has been associated with increased reactive oxygen species (ROS) generation, which can cause oxidative damage to lipids, proteins, and DNA, ultimately leading to apoptosis or necrosis [33,55]. It is worth noting that at physiological levels of shear stress, apoptosis can be inhibited in human endothelial cells, indicating that cellular responses depend on the magnitude and duration of the applied stress [56].

When temperature-sensitive bioinks are used, thermal stress can induce protein unfolding and the accumulation of non-native proteins, which activate heat shock responses and related signaling pathways. Even under moderate temperature changes, these responses reflect cellular adaptation to thermal perturbations and may influence cellular function and survival [36,57]. In addition, when photocrosslinkable bioinks are employed, UV-induced damage can result in DNA strand breaks and genomic instability, which may activate DNA damage responses and apoptosis pathways, thereby affecting cellular function and survival [37]. For example, UV exposure during hydrogel photocrosslinking has been reported to reduce the viability of HepG2 cells (human hepatocellular carcinoma–derived liver cell line) at UV irradiation doses above 1350 mJ cm^−2^ [39]. The magnitude of mechanical, thermal, and radiative stresses can be controlled through changes in parameters such as nozzle geometry, extrusion pressure, printing temperature, and UV light exposure [41,42,43,44].

In stereolithography-based 3D printing, UV or visible light exposure can induce cellular damage through photochemical effects, including reactive oxygen species (ROS) generation and DNA damage [38,58,59]. Such damage may activate intracellular signaling pathways associated with stress responses and apoptosis, ultimately affecting cell viability and function [37]. For example, UV doses above 0.5 kJ m^−2^ affect the cell viability of fibroblasts and mesenchymal stem cells [53]. Stereolithography-based 3D printing can be used to study cells’ responses to localized and transient radiation-driven extreme environments. The magnitude of radiative stress can be controlled by changing parameters such as light intensity (UV or visible), exposure duration, irradiation energy, and photoinitiator properties [38].

It has been reported that in laser-assisted bioprinting, laser exposure at wavelengths such as 355 nm and 532 nm, with fluence in the range of approximately 330–850 mJ cm^−2^, can affect cell viability due to combined photochemical and photothermal effects [60]. Such exposure can generate reactive oxygen species (ROS) and induce DNA damage, leading to alterations in cellular function and survival [37,60].

### 2.3. Limitations of the Approach

There are several limitations of this approach. During printing, cells are exposed to mechanical, thermal, or radiative stresses that are localized and transient, occurring primarily within confined regions such as the nozzle or light exposure zone and lasting for short durations. As a result, these conditions mainly reflect the acute stresses that cells experience. Cells’ responses include immediate viability loss or oxidative stress [34,61]. The extreme environments human bodies experience typically involve prolonged exposure that enables physiological adaptation through coordinated biochemical and tissue-level regulation. Due to the lack of sustained environmental exposure, this approach is unable to evaluate long-term environmental adaptation mechanisms. Although having these limitations, studies using this approach can result in new insights into human cells’ responses to extreme environments where the duration is short (transient) and the stress is localized.

## 3. Investigation of Cells’ Responses to Extreme Environments Using 3D Printed Samples

### 3.1. Fabrication of 3D Constructs (Embedded with Cells) by 3D Printing

Bioprinting enables cells to be encapsulated within 3D microenvironments that more closely resemble those in native tissues [62,63]. Although bioprinted constructs do not replicate full organ-level or systemic physiology, they enable controlled investigation of cellular-level responses to specific aspects of extreme environments by changing construct design parameters—such as bioink composition, construct geometry, and concentration of cells in printed constructs.

The type of bioink determines the baseline biochemical and structural environment surrounding the cells embedded in the bioink. Different bioinks, such as alginate, PEGDA, hyaluronic acid, chitosan, fibrin, silk fibroin, gelatin, agarose, methylcellulose, and collagen, have distinct properties that influence cell–matrix interactions, adhesion, and signaling [64]. Increasing polymer concentration creates a stiffer matrix [65], which can be used to model mechanical stress environments, where cells experience higher resistance and force transmission. In contrast, using softer or more porous bioinks improves permeability and mass transport [61], enabling the study of diffusion-related conditions, such as localized hypoxia or nutrient limitation.

The geometry of printed constructs directly influences diffusion gradients, leading to spatial variations in oxygen and nutrient availability for the cells embedded in printed constructs. Thick or densely printed constructs often develop hypoxic cores, which can alter cell viability, metabolic activity, and stress responses [66,67]. Reported studies have shown that such gradients can be intentionally leveraged to model oxygen-limited environments and investigate hypoxia-induced cellular behavior [68]. Additionally, constructs can be printed in a way that different portions of the constructs can have different oxygen levels. Exposing such constructs to externally applied extreme environments can reveal cellular responses in these different portions of the constructs. In several reported studies, mammalian cells have been co-printed with photosynthetic microalgae that produce oxygen within the constructs and help regulate local oxygen availability [69,70].

### 3.2. Exposure of Printed Constructs to Different Environmental Stressors by Using Different Test Chambers

Bioprinting can be used to study cells’ responses to extreme environments by placing bioprinted 3D constructs into environmental testing chambers. Following printing, customized 3D constructs can be transferred into temperature-controlled systems, hypoxia workstations, pressure chambers, or other defined environmental platforms. This approach allows controlled exposure to specific environmental stress condition. For instance, Rahman et al. investigated the effects of thermal stress (37 °C, 45 °C, and 55 °C for 10 min) on HBE cells embedded in 3D printed constructs [71]. In this case, the extrusion-based platform provided a reproducible three-dimensional tissue model, while the thermal stress was applied post-printing as a separate environmental variable using a heat block.

### 3.3. Limitations of the Approach

Despite the advantages of 3D printed constructs for studying cells’ responses under defined post-printing environmental conditions, these printed constructs are subject to fundamental limitations related to mass transport within hydrogel matrices. In most printed constructs, oxygen, nutrients, and metabolic waste are transported primarily through passive diffusion. Because bioprinted hydrogels typically lack perfusable vasculature, oxygen supply to cells depends largely on diffusion through the surrounding matrix. The extent of diffusion limitation therefore depends on several experimental parameters, including construct thickness, cell density, and hydrogel properties such as porosity, polymer concentration, crosslinking density, and water content [66]. For example, as construct thickness or cell density increases, cellular oxygen consumption may exceed the rate at which oxygen diffuses through the matrix. In hydrogel-based constructs, oxygen diffusion is generally limited to approximately 100–200 µm from the construct surface, beyond which oxygen delivery may become insufficient to sustain cellular metabolism [72]. Consequently, cells located deep inside of thick constructs may experience hypoxia even under incubator conditions with normal oxygen concentration.

These diffusion-induced gradients of oxygen level can complicate the interpretation of results from experiments designed to study cells’ responses to externally applied extreme environments. For example, when studying hypobaric or hypoxic conditions, oxygen deprivation may arise not only from the imposed environmental conditions but also from intrinsic diffusion limitations within the construct. It may therefore be difficult to distinguish between environmentally induced hypoxia and diffusion-driven oxygen depletion. Moreover, due to diffusion constraints, the absence of functional vascular networks, and the lack of systemic physiological regulation, this approach remains limited in their ability to reproduce complex tissue-level and organism-level responses to extreme environments.

Several strategies have been reported to mitigate diffusion-induced gradients of oxygen and nutrient level in three-dimensional constructs. One strategy is the incorporation of vascular channels or perfusable microvascular networks, which enable convective transport of oxygen and nutrients within the construct [73]. This is critical because oxygen and nutrient delivery in bioprinted tissues rely largely on diffusion in the absence of a functional vascular network. Another strategy is dynamic culture systems, such as perfusion bioreactors or microfluidic platforms, which can further improve mass transport by introducing controlled fluid flow [74,75]. An additional strategy is limiting construct thickness as thin constructs (≤200–300 µm) that are less susceptible to diffusion limitations, whereas thicker constructs (>500 µm) are more prone to central hypoxia unless active transport mechanisms are introduced [76]. Finally, proper hydrogel design, including increased porosity or optimized crosslinking density, can improve molecular diffusion within the matrix [73].

## 4. Concluding Remarks

Bioprinting can be used for studying human cells’ responses to extreme environments. First, localized and transient extreme environments can be intentionally created during the printing process. The intensity of the extreme environments can be varied by adjusting printing parameters (such as nozzle geometry, extrusion pressure, printing speed, temperature, or light exposure). This approach enables the investigation of cells’ responses to localized and transient mechanical, thermal, or radiative stresses. However, because these stresses are localized and transient, they do not represent long-duration environmental exposure. This paper summarizes, for the first time in the literature, publicly available information in the use of 3D printing to create extreme environments during 3D printing to study cells’ responses to localized and transient extreme environments. In some relevant papers, this use of 3D printing might be implied but is not explicitly stated.

Second, bioprinting enables fabrication of 3D cell-laden constructs that better mimic native tissue architecture. These 3D constructs can be exposed to extreme environments under controlled conditions and can contain integrated sensors that allow real-time monitoring of cellular responses. However, because bioprinted constructs lack full vascularization, perfusion, and systemic physiological interactions, the results obtained using these constructs might not be directly translatable to whole-body human responses. This paper summarizes, for the first time in the literature, publicly available information on the use of 3D printed constructs to investigate the responses of cells (embedded in the printed constructs) to extreme environments. In some relevant papers, this use of 3D printing might be implied but is not explicitly stated.

## Figures and Tables

**Table 1:** Several reported studies on human body’s responses to extreme environments.

Extreme Environment	Test Model	Response	Reference
Heat	Human, male and female	Transcriptomic response of peripheral blood mononuclear cell	[18]
Hypobaric	Human, male trekker	Acute mountain sickness evaluation, electrocardiograph, extravascular lung water accumulation by thoracic ultrasound, middle cerebral artery blood flow velocity, muscle and cerebral oxygenation	[13]
Hyperbaric	Human, male divers	Oral bacterial metabolism, bacterial oxidative stress response	[19]
Hyperbaric	Human, male divers	Blood cell counts, cardiac damage, oxidative stress, vascular endothelial activation, and hormonal biomarkers	[20]

**Table 2:** Several reported studies on cells’ responses to extreme environments.

Extreme Environment	Test Model	Response	Reference
Heat	Mouse neural stem cells	The number of adherent cells, expression ratios of HS protein (Hsp)40and Hsp70genes	[21]
Heat	Human mesenchymal stem cells	Metabolic activity and viability	[25]
Simulated microgravity	Mouse primary T cells, Human T lymphocytes cells	T-cell transcriptome analysis using RNA sequencing	[22]
Spaceflight microgravity	Human bone marrow mesenchymal stem cells	Transcriptomic response via RNA sequencing	[23]

**Table 4:** Reported studies on cells’ responses to localized and transient extreme environments created during 3D printing.

Bioprinting Technique	Printing Parameter	Range	Cell Type	Result	Reference
Extrusion-based	Nozzle diameter	150–400 µm	HepG2 cells	Decreased cell viability as nozzle diameter decreased	[41,42]
	Extrusion pressure	0.5–5 bar	HepG2 cells, hiPSCS cells, human skin fibroblast cells	Decreased cell viability and increased membrane damage as extrusion pressure increased	[41,42,43,44]
	Nozzle length variation	8.9–24.4 mm.	Human pluripotent stem cells (hPSCs)	Decreased cell viability as nozzle length increase	[44]
	UV-A irradiation dose	1350–5400 mJ cm^−2^	HepG2 cells	Reduced viability with prolonged exposure (phototoxicity)	[41]
Inkjet-based	Pulse amplitude	40–80 V	Human fibroblasts cells	Decreased cell viability as pulse amplitude increased	[52]
Laser-assisted	Laser fluence	800–1600 mJ cm^−2^	NIH 3T3 mouse fibroblast cells	Decreased cell viability as the laser fluence increases	[39]
Light-based bioprinting	UV dose	0.5–20 kg/m^2^	L929 mouse fibroblasts, human mesenchymal stem cells	Induced DNA damage and increased apoptosis under higher UV intensity/exposure conditions	[53]

## Data Availability

The authors confirm that the data to support the findings of this study are available within the article or upon request to the corresponding author.
